# *TP53* Mutations in Acute Myeloid Leukemia: Still a Daunting Challenge?

**DOI:** 10.3389/fonc.2020.610820

**Published:** 2021-02-08

**Authors:** Matteo Molica, Carla Mazzone, Pasquale Niscola, Paolo de Fabritiis

**Affiliations:** ^1^ Haematology Unit, S. Eugenio Hospital, ASL Roma 2, Rome, Italy; ^2^ Department of Biomedicina and Prevenzione, Tor Vergata University, Rome, Italy

**Keywords:** acute myeloid leukemia, *TP53* mutations, poor outcome, decitabine, venetoclax (BCL-2 inhibitor)

## Abstract

*TP53* is a key tumor suppressor gene with protean functions associated with preservation of genomic balance, including regulation of cellular senescence, apoptotic pathways, metabolism functions, and DNA repair. The vast majority of *de novo* acute myeloid leukemia (AML) present unaltered *TP53* alleles. However, *TP53* mutations are frequently detected in AML related to an increased genomic instability, such as therapy‐related (t-AML) or AML with myelodysplasia-related changes. Of note, *TP53* mutations are associated with complex cytogenetic abnormalities, advanced age, chemoresistance, and poor outcomes. Recent breakthroughs in AML research and the development of targeted drugs directed at specific mutations have led to an explosion of novel treatments with different mechanisms. However, optimal treatment strategy for patients harboring *TP53* mutations remains a critical area of unmet need. In this review, we focus on the incidence and clinical significance of *TP53* mutations in *de novo* and t-AML. The influence of these alterations on response and clinical outcomes as well as the current and future therapeutic perspectives for this hardly treatable setting are discussed.

## Introduction

**Figure 1:**
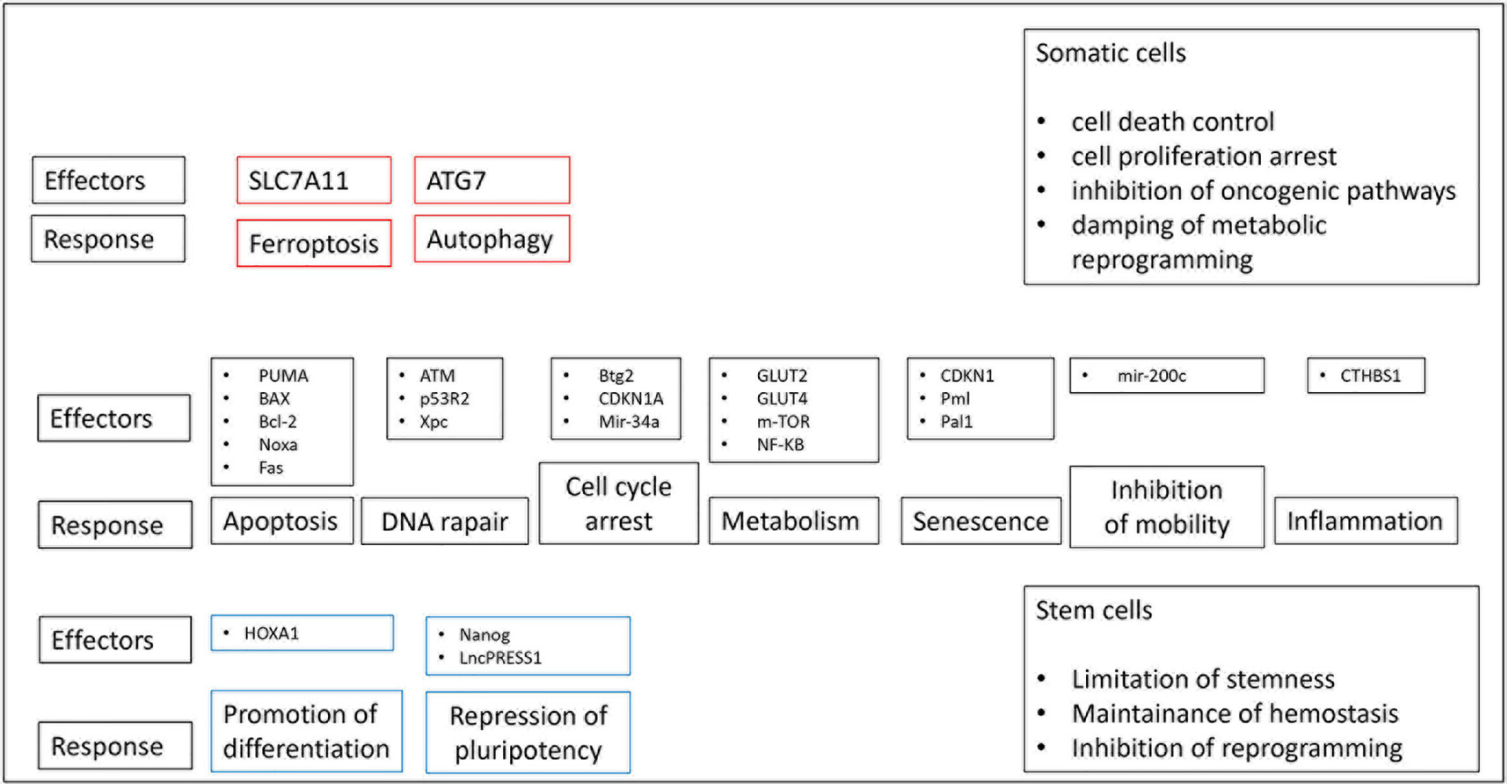
Several p53-mediated pathways potentially affect upon a common result of tumor suppression. P53 has the capability to control common and different biological pathways in somatic and stem cells. P53-mediated biological pathways in red boxes are specific to somatic cells, those in the blue boxes are specific to stem cells and those in black boxes are common to both cell types.

The tumor suppressor gene *TP53* encodes a transcription factor, which operates after the stimulation of various forms of cellular stress and, employs multiple anti-proliferative functions (**Figure 1**). The transcription factor includes a DNA binding domain, a transcription activation domain, a tetramerization domain and a proline-rich domain (1). The most well‐known property of *TP53* gene is its capability in containing cellular proliferation/differentiation associated with aberrant and uncontrolled oncogene expression. Therefore, *TP53* inactivation inducing by gene mutation or deletion favors the activities of oncogenes, thereby promoting uncontrolled proliferation of cancer cells (2). Conversely to the vast majority of tumor suppressor genes that are predominantly characterized by truncating mutations, a substantial proportion of *TP53* alterations include missense substitutions (75%); other mutations are frameshift insertions and deletions (9%), non-sense mutations (7%), silent mutations (5%), and other rare aberrations (2%). While wild-type (WT) p53 tends to prevent a tumorigenic phenotype, either loss-of-function or oncogenic gain-of-function (GOF) *TP53* mutations favor tumorigenesis events. The majority of the gene alterations detected in patients with hematological diseases include GOF mutations. For instance, *IDH1/2* gene acquires a novel function to convert α-ketoglutarate (α-KG) to 2-hydroxyglutarate (2-HG) determining the inhibition of TET2 as well as some histone demethylase and promoting tumorigenesis (3). *TP53* gene represents the most commonly altered gene in somatic cells of human malignancies, with mutations in *TP53* gene detected in more than 50% of solid tumors (4–6). In some tumors, such as colorectal cancer, *TP53* alteration is frequently a belated occurrence in a multi-stage carcinogenic pathway that proceeds from hyper-proliferative cells in colonic epithelium to colorectal adenomas and ultimately to metastatic colorectal cancer (7, 8). Contrariwise, in high-grade serous ovarian tumors, *TP53* mutations are a relatively precocious event, likely arising in forerunner lesions (9, 10). Germline *TP53* mutations may cause Li-Fraumeni disease; carriers tend to develop several cancers including early onset brain and adrenocortical tumors, breast cancer, soft tissue sarcoma, and leukemia (11). In the last decade, robust discoveries have been made exploring the mutational landscape of hematological diseases mainly thanks to developments in sequencing techniques. The recent progress of next-generation sequence (NGS) has increasingly included it in clinical research allowing the cytogenetic analysis in several hematological malignancies, including acute myeloid leukemia (AML) (12). Therefore, to date, NGS is the most common and reliable method used to detect *TP53* mutations in AML, holding higher sensitivity than other techniques such as immunohistochemistry (IHC), fluorescent *in situ* hybridization (FISH), or real-time qualitative reverse transcription PCR (RT-qPCR). Despite the frequency of *TP53* mutations detected in human cancers, they are observed in only 5–10% of patients with AML. Although all classes of *TP53* variants have been observed in AML, the vast majority of mutations include missense alterations usually arising in the DNA binding domain (encoded by exons 5–8) with a predilection for arginine residues and noted mutational “hot spots” such as R175H, Y220C, R248Q, and R273C (13). Interestingly, despite variants in *TP53* usually determined a loss-of-function of the tumor-suppressor activity of the protein, the R282 site was found to belong to the gain-of function (GOF) mutations (14). R282 represents a structural mutation (as also e.g. R175, G245, R248, und R249) inducing conformational instability of the p53 protein, by contrast to the contact mutations (R273 and R248) that are sited in the p53-DNA binding surface (15). Mutations in R282 were only observed in the acute diseases AML and acute lymphoblastic leukemia (ALL) and might accelerate the rapid proliferation of the malignant cells in these diseases (14, 16). The single p53 deletion caused by loss of 17p chromosome in also included among *TP53* mutations. A deletion of 17p commonly involves the tumor suppressor gene p53 on band 17p13.1 with allelic loss of the gene (17). It was widely demonstrated that p53 deletion without multiple cytogenetic aberrations is an independent negative prognostic factor for disease-free survival (DFS), relapse risk, and overall survival (OS) in AML (17). Therefore, the single p53 deletion should be always considered as a high-risk aberration for new risk-adapted treatment strategies in AML. Quintás-Cardama et al. have recently reported that, in addition to somatic mutations, p53 dysfunction can occur *via* aberrant expression of proteins that regulate p53 stability and function (e.g. overexpression of its canonical negative regulators Mdm2 and/or Mdm4) (18). They demonstrated that reactivation of p53 functions *via* Mdm2-antagonists in the context of Mdm2 overexpression and wild-type p53 restored p53’s anti-tumor effects. These data suggest that specific recommendations could be considered in patients with AML expressing p53 protein stabilization and to those exhibiting Mdm2 protein overexpression. In the latter case, a strong recommendation to enroll in trials assessing experimental agents should include those with Mdm2 blocking activity. Mutations in other functional domains hold specific effects on p53 protein activities; however, to date, their clinical implications in AML are not well understood (19). *TP53* is the sole mutated gene detected in up to 75% of patients, while patients who harbor co-occurring mutations show a lower incidence of mutations in several AML-related genes such as *NPM1*, *FLT3, IDH1, IDH2, WT1, DNMT3A, RUNX1*, and *RAS* (20, 21). The median variant allele frequency (VAF) has been reported to be the highest of all recurrently mutated genes in AML at nearly 50%, with a higher VAF potentially predictive of inferior survival in MDS and AML patients (22). *TP53* alterations are associated with higher age in AML and ALL, while no such difference was found in chronic lymphocytic leukemia (CLL) and myelodysplastic syndromes (MDS) patients. Notably, this effect was specifically detected for *TP53* mutations but not for *TP53* deletions. It can be suggested that *TP53* alterations seem to have a higher impact in acute diseases (AML, ALL) compared to MDS and CLL patients, as they are more frequent and show a clear increase with age (23).

## Significance and Incidence of *TP53* Mutations in *De Novo* Acute Myeloid Leukemia (AML)

**Figure 2:**
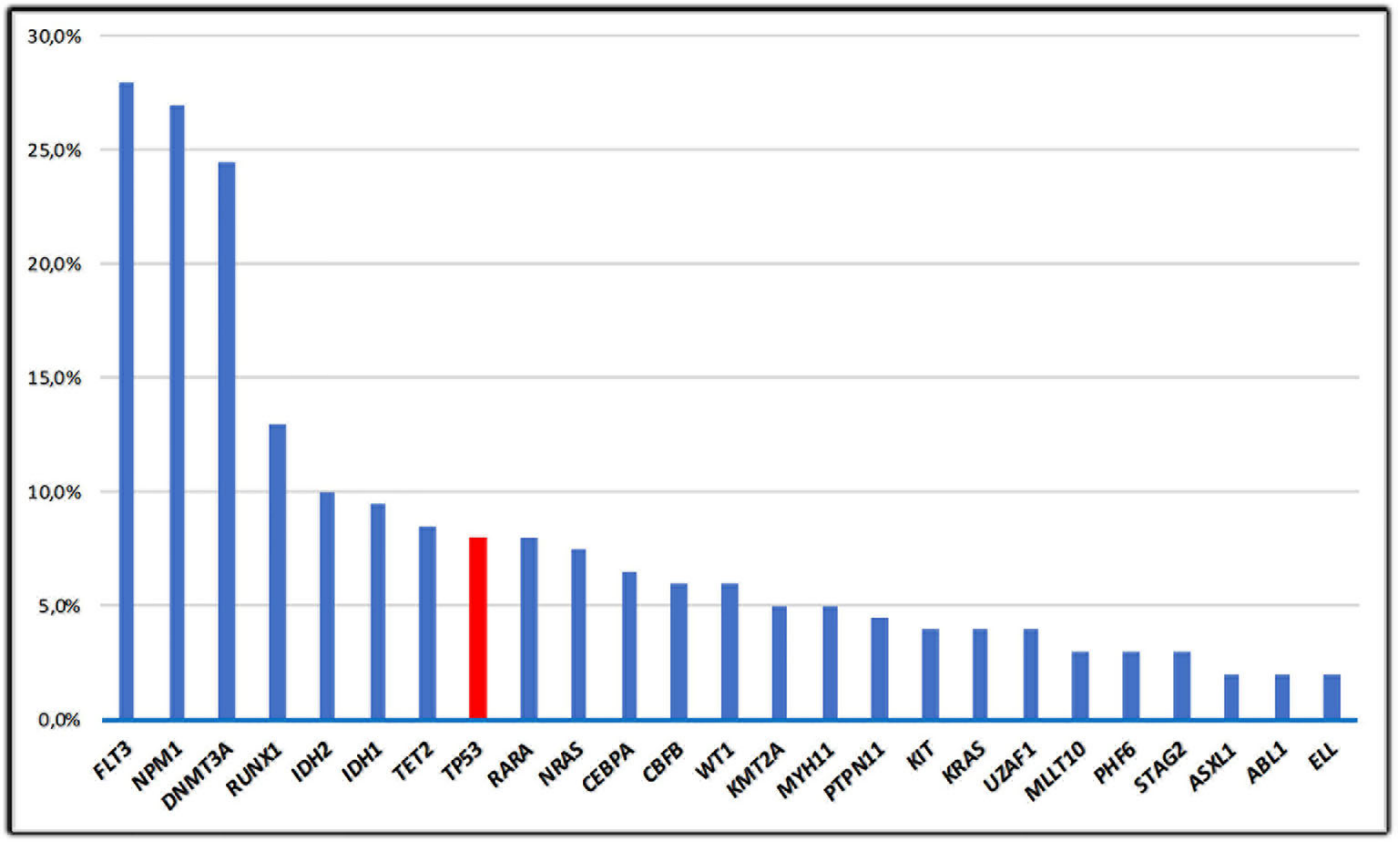
The frequency of TP53 mutations in *de novo* AML; mutations in AML from the TCGA study (11) are shown in this figure. Only the 25 most frequent mutations are represented.

The vast majority of cases of *de novo* AML have unaltered *TP53* alleles; data from The Cancer Gene Atlas (TCGA) including adult patients with AML documented that ~8% of AML cases harbor *TP53* mutations (24, 25) (**Figure 2**). A consistent proportion (70–80%) of *TP53* mutations in *de novo* AML includes missense substitutions causing amino-acid changes; the remaining mutations are truncating alterations. Missense changes determine a prolonged half‐life of the altered proteins, compared with its wild‐type, short‐lived counterpart. The molecular mechanisms leading to the prolonged half-life of mutant *TP53* protein are not still completely clarified. It could in part be associated with the incomplete degradation produced by the E3 ubiquitin ligase Mdm2, whose gene is considered as a selected transcriptional target of WT-*TP53*. Consequently, its activation is impaired in cells carrying mutant *TP53* proteins (26). The vast majority of mutations recurrently earmark “hot-spot” codons. Some “hot-spot” codon positions such as Y220C, R248Q, P72R, R273C, and R175H often overlap between AML-related gene mutations and *TP53* alterations detected in solid malignancies (2, 4). These alterations either directly produce the degradation of the DNA-binding domain of *TP53* or induce conformational variations of the *TP53* protein, thus determining a sever impairment of *TP53* functions (27). Analysis of the respective frequency of *TP53* mutations across the TCGA datasets shows that AML is one of the tumors with the lowest *TP53* alteration rate among all human cancers (24). This may mean that both *TP53* inactivation is not necessary for the occurrence or maintenance of AML and AML may influence *TP53* activities due to alternate mechanisms. In AML, the loss of one allele of chromosome 17p is frequently associated with *TP53* mutation resulting in a loss of heterozygosity (25). In contrast, *TP53* mutations unrelated to cytogenetic alterations represent a rare event (28). In addition, 10–30% of *de novo* AML cases harboring *TP53* mutations hold a cytogenetically detectable *TP53* deletion with wild-type conformation of the remaining allele (29). Results from studies conducted on mice show that the loss of one *TP53* allele might be sufficient for promoting multistep leukemogenesis mechanisms (30). This may be significant for the occurrence of leukemia in patients harboring a single *TP53* deletion. An alternative hypothesis consists in the inhibition of downstream mediators involving in the *TP53* intracellular pathways, which affect both the cell-cycle arrest and the repair of DNA damages and mechanism of apoptosis. Alternatively, up-regulation of genes, which inhibit *TP53* functions and favor inactivation of *TP53* may be contemplated, for instance *MDM2* gene amplifications have been identified in CLL (31). Aberrant overexpression of the p53 protein is usually associated with *TP53* alterations and a complex karyotype, but the prevalence and impact of p53 overexpression in AML with diploid cytogenetics is unknown. Assi et al. analyzed survival outcomes according to p53 expression level quantified in bone marrow core biopsy samples using immunohistochemistry and computer‐assisted image analysis, in patients with diploid karyotype. They observed that the median leukemia free survival was significantly shorter in the subset of patients with p53 overexpression (9 *vs* 55 months; p = 0.026), including those who were consolidated with allogeneic stem-cell transplantation (ASCT) (32). These data suggested that p53 expression level could be helpful to stratify patients with AML and a diploid karyotype, currently classified as intermediate‐risk disease and that p53 expression level could help to identify patients who could possibly benefit from post-ASCT maintenance therapy. Several reports have also described the association between erythroid/myeloid leukemia or AML-M6a (acute erythroid leukemia [AEL]) with high-risk biological features, including high frequency of *TP53* mutations in up to 53% of patients with AEL (33, 34). Recently, Montalbano-Bravo et al. showed that also in pure erythroid leukemia (PEL) which represents 14% of cases of all erythroid leukemia, there is a high prevalence of chromosome 17 abnormalities and mutations in *TP53*; interestingly, in several patients, there was co-occurrence of both, or double *TP53* mutations. Therefore, these data demonstrated that the loss of *TP53* function was a strong influential factor for PEL pathogenesis consisting in dismal prognosis irrespective of currently available therapies and that the enrolment in clinical trials targeting or circumventing mutant p53, and less intensive approaches is the current optimal strategy for this subset of patients (35).

## *TP53* Co-occurring Mutations in AML

Assessment of co-occurring gene mutations has identified *TP53*-altered AML cluster as a unique disease subgroup; nevertheless, AML with *TP53* mutations are not specifically correlated with a unique transcriptional signature by standard RNA sequencing. In fact, *TP53* alterations may co-exist with some other frequent AML-related single-nucleotide mutations such as *TET2*, *NPM1, FLT3, DNMT3A, IDH1*, and *IDH2*; in addition, *TP53-*mutant AML are often characterized by recurrent co-occurring karyotypic structural aberrations, especially genomic abnormalities detected in certain chromosomes (chromosome 5, 7, and 17), and with events involving chromothripsis (36–38). Interestingly, although karyotypic instability has been associated with *TP53* inactivation, only distinct forms of increased mutagenesis have been identified among *TP53-*mutated AML; *TP53-*mutations tend to co-exist with augmented numbers of major karyotipic abnormalities, marker chromosomes and chromothripsis (39), but not with a potentially rise in single-nucleotide mutations (40). Mutations can co-occur within founding clones or sub-clones or can be found in alternative clones, which are not correlated with the neoplastic hematopoietic cell. Two patterns of *TP53* co-mutations often emerged. First, mutations in *TP53* and epigenetic genes (i.e. *IDH1*, *IDH2*, *DNMT3A*, *TET2*) and transcription factors (i.e. *CEBPA*, *RUNX1*, *NPM1*) commonly co-occur in the founding clone. Contrariwise, mutations involving triggered signaling pathways (i.e. *FLT3*, *RAS*, *PTPN11*, *BCOR*, *JAK2*, *NF1*) and polycomb mechanisms (i.e. *SF3B1*, *KDM6A*, *SRSF2*) frequently occurred in sub-clones, indicating that these alterations may productively stimulate *TP53* AML throughout clonal evolution. Mutations involving *ASXL1*, *CBL*, and *U2AF1* genes arise almost identically in sub-clones of *TP53*, and with *TP53* as a variant in a novel incoming sub-clone (36). Kadia et al. analyzed peripheral blood or bone marrow samples from 293 *de novo* AML patients using targeted amplicon-based NGS-based mutation analysis. They found *TP53* mutations in 53 patients (18%); 45 patients harbored a missense mutations with the most frequent amino acid replacement being a substitution of arginine for histidine in various codons. There was a significant lower presence of mutations involving *FLT3* (6 *vs.* 19%; p = 0.02), *RAS* (4 *vs.* 14%; p = 0.04), and *NPM1* (8 *vs.* 20%; p = 0.03) genes in mutant-*TP53* AML in comparison with WT-*TP53* AML (41). Mutations of *TP53* were detected by NGS in 105 out of 2,272 (5%) AML patients enrolled in the Study Alliance Leukemia AML96 and AML2003 multicenter studies. Among these patients, none showed additional low-risk mutations, such as t(8;21) or inv (16), while a significant direct correlation between *TP53* deletion and other high-risk features was observed for del(5q) (*P* < 0.001), −5 (*P* < 0.001) and −7 (*P* < 0.05). The well-known molecular risk factors *FLT3-ITD* (internal tandem duplication) and *NPM1* mutation showed a lower incidence then *TP53* deletion in patients with complex karyotype (*P* < 0.001) (17). An Italian study analyzed 40 AML patients for copy number alterations (CNAs) (detected by Affymetrix SNP arrays) potentially associated with *TP53* and found several CNAs ranging from loss to gain of complete chromosome arms. Specifically, gains located on chromosome 8 were statistically associated with *TP53* mutations. In addition to trisomy of chromosome 8, others CNAs related to other chromosomes are significantly associated with *TP53* mutations: loss of chromosome 5q, deletion of chromosome 3(p22.3), deletion of chromosome 12(p12.3), and gain of chromosome 17(p11.2), chromosome 16p(11.2-11.3), and chromosome 14(q32.33) (42). Recently, the International Working Group for Prognosis in MDS (IWG-PM) assessed 3,324 MDS patients for *TP53* mutations and allelic imbalances of the *TP53* locus observing that one in three patients harboring *TP53* mutations displayed mono-allelic targeting of the gene whereas two in three showed multiple hits reflecting bi-allelic targeting. The noted correlation between *TP53* and high-risk presentation, complex karyotype, rapid leukemic transformation, and poor survival were directly associated with patients displaying the multi-hit state only. *TP53* multi-hit state resulted in combination with high-risk occurrence, complex karyotype, and dismal survival and predicted risk of leukemic transformation and death regardless of the Revised International Prognostic Scoring System, while patients presenting mono-allelic alterations did not show differences when compared with those with WT-*TP53* (43). In AML, *TP53* mutations correlate with low-rate of response to standard chemotherapy regimens and dismal OS in patients presenting or not a complex karyotype. By using a multivariate analysis, the presence of *TP53* mutations without a cytogenetic aberration predicted for inferior OS and lower response to chemotherapy (26). In fact, all patients with *TP53* deletion showed significantly poorer 2-year DFS as well as shorter 2-year OS compared to patients with complex aberrancy without *TP53* deletion (29). Furthermore, 17p-loss of heterozygosity (LOH) (44), chromosome 17 abnormalities (45), and up-regulation of full-length protein isoform 22 (46) additionally predicted for unfavorable prognosis in AML. Detection of the *TP53* allelic state is crucial for defining diagnosis, risk assessment and precise prognostication in MDS, and further studies should include the evaluation of *TP53* allelic state in AML. *TP53* mutations were assessed not only as a dichotomous variable. With the advent of NGS technologies, mutational sub-clones can now be detected with high sensitivity. A recent study investigated the clinical characteristics associated with sub-clonal *TP53* mutations and their prognostic impact in a large cohort of AML patients prospectively treated within studies of the “German-Austrian AML Study Group” (AMLSG). A total of 108 *TP53* mutations were found in 98 patients (6.4%) and patients with *TP53* mutations were categorized by their VAF into groups with frequencies >40, 20–40, and <20%. Authors demonstrated that even *TP53*-mutated sub-clones, defined by a VAF <20%, have a statistically significant negative prognostic impact with respect to complete remission rate, OS, and EEFS. However, compared to patients who harbored clonal *TP53* mutations, those with sub-clonal alterations showed significantly fewer complex karyotypes and chromosomal losses (47). These data suggest that sub-clonal *TP53* mutations represent a novel significant prognostic factor in AML and may have implications for incorporating NGS among *TP53* routinely screening methods and for re-defining future risk stratification in AML.

## Role of *TP53* Mutations in Therapy-Related AML (t-AML)

Mutations involving *TP53* gene are more commonly found in AML characterized by augmented genomic instability, such as relapsed AML, therapy-related (t-AML), and older AML patients (48, 49). In t-AML, *TP53* mutations are present in about 30% of patients (**Table 1**) (44), while in elderly patients with complex‐karyotype AML, *TP53* lesions can be present in up to 70% of cases (57). Over the last years, it was hypothesized that the higher rate of *TP53* mutations in t-AML was directly associated with the cytotoxic effect of chemotherapy and radiation on hematopoietic stem/progenitor cell (HSPC) DNA. However, recently Wong et al. reported that the cytotoxic effects of standard chemotherapies do not directly produce *TP53* mutations. They postulated a model in which a low number of hematopoietic stem/progenitor cells (HSPC) (<0.7%) harboring age-related *TP53* mutations present resistance to conventional therapies and expand preferentially after chemotherapy (54). The precocious occurrence of *TP53* genetic alterations in the founding HSPC clones likely determines the increased rate of cytogenetic aberrations and scarce responses to treatments that are typically observed in t-AML/t-MDS patients. It is thus challenging to predict which cases may develop a t-AML and to find ways to prevent it. An MD Anderson Cancer Center (MDACC) study, which collected 108 consecutive patients with t-MDS/t-AML showed that *TP53* mutations in this setting were predominantly located in DNA-binding domains presenting an allelic frequency of 37% (range between 7.1 and 98.8). The majority of *TP53* gene mutations included single nucleotide variants, among which transitions (65.9%) were more frequently observed than transversions (34.1%). Missense mutations appeared the most commonly detected, followed by non-sense and frameshift alterations and the *TP53* mutation pattern was notably similar to that observed in newly diagnosed MDS/AML (29). Peculiar subsets of altered genes detected in t-AML and in newly diagnosed AML have been reported. According to prior studies (41, 44, 45), *TP53* mutations were remarkably higher in t-AML compared with *de novo* AML but presented lower rate of driver mutations such as *NPM1* and *DNMT3A*. Furthermore, *TP53* mutations seem to be mutually exclusive with other gene mutations such as *NPM1*, *FLT3*, *ARF*, and *MDM2* (58).

**Table 1:** Recurrent mutations in therapy-related neoplasms.

Gene mutation	Therapy-related myeloid neoplasms frequency (%)	References
**ASXL1**	3–17	Lindsley et al. (50)
**CEBPA**	0–5	Christiansen et al. (51)
**DNMT3A**	8–27	Lindsley et al. (50); Shih et al. (52)
**EZH2**	3–4	Lindsley et al. (50); Shih et al. (52)
**FLT3**	8–16	Lindsley et al. (50); Christiansen et al. (51)
**IDH1**	3–5	Voso et al. (53); Wong et al. (54)
**IDH2**	0–5	Voso et al. (53); Wong et al. (54)
**KMT2A**	3	Bacher et al. (55)
**KRAS**	11	Lindsley et al. (50)
**NPM1**	4–16	Lindsley et al. (50); Wong et al. (54)
**NRAS**	10–13	Lindsley et al. (50); Bacher et al. (55)
**PTPN11**	3–9	Wong et al. (54); Christiansen et al. (56)
**RUNX1**	11–16	Lindsley et al. (50); Christiansen et al. (51)
**SF3B1**	0–3	Lindsley et al. (50); Shih et al. (52)
**SRSF2**	8–11	Lindsley et al. (50); Shih et al. (52)
**TET2**	6–14	Lindsley et al. (50); Shih et al. (52)
**TP53**	23–37	Lindsley et al. (50); Shih et al. (52); Wong et al. (54)
**U2AF1**	5–8	Lindsley et al. (50); Wong et al. (54)

## Prognostic Impact of *TP53* Mutations in AML

The European Leukemia net (ELN) guidelines represent probably the most established source for evaluating risk of resistance and classifying patients into “favorable,” “intermediate,” and “unfavorable” groups according to cytogenetic and molecular mutations detected in leukemic cells. The 2017-updated version considers *TP53* mutated cases as an independent group to be included in the adverse prognostic risk category (59). In 2012, the German group identified five distinct prognostic subgroups according to molecular characterization through a univariate and multivariate Cox regression analyses (60). *TP53* mutated cases treated with intensive standard chemotherapy [“3+7” scheme, cytarabine (ARA-C)-daunorubucin (DNR) based regimen] were a very unfavorable prognostic group presenting the worse outcome (OS at 3 years: 0%). Recently, Papaemmanuil et al. analyzed 1,540 patients with AML using targeted sequencing of 111 myeloid cancer genes (13). Patterns of co-mutations distinguished AML patients into 11 categories, each with a specific clinical behavior and outcome. In addition to noted disease classes, three more heterogeneous AML categories were identified: AML presenting mutations of chromatin and RNA-splicing regulators, AML harboring *TP53* mutations and/or chromosomal aneuploidies, and AML with *IDH2* ^R172^ abnormalities. Patients with chromatin–spliceosome and *TP53*–aneuploidy AML presented dismal prognosis, with the different class-defining mutations independently and additively affecting outcomes. Multiple studies have reported that the presence of *TP53* mutations produce low response to treatments, significant rate of relapses, and poor prognosis (60, 61). Using a multivariate analysis adjusted for age, performance status, cytogenetic risk group, *de novo*/t-AML leukemia, and white blood cell count, Bowen et al. demonstrated that having a *TP53* mutation was independently correlated with inferior survival in adult AML patients treated with ARA-C-anthracycline based treatments (DAT = DNR, ARA-C, thioguanine and ADE = DNR, ARA-C, etoposide regimens) (62). Outcome results of patients presenting high-risk cytogenetic score at diagnosis showed that the presence of *TP53* mutations contributed to an inferior rate of complete response (CR) (28% mutated *versus* 50% un-mutated), and a lower DFS and OS (59). Although ASCT represents the unique potentially curative treatment for *TP53*-mutated AML/MDS patients, the risk of death after the procedure remains significantly high, with the majority of deaths mostly related to the occurrence of disease relapse. In a recently published analysis of the European Society for Blood and Marrow Transplantation (EBMT), authors reported that patients with a 17p abnormality who achieved a CR1 and underwent ASCT had a 2-year OS and leukemia-free survival rate of 28 and 24%, respectively. The 2-year non-relapse mortality (NRM) was 15%, and the 2-year rate of relapse was 61% (63). Among a large cohort of 289 patients with *TP53-*mutated MDS assessed in a Center for International Blood and Marrow Transplant Research study, the 3-year OS was 20%, while the median OS was 0.7 years (64). The MDACC group postulated that the outcome of *TP53*-mutated AML/MDS patients are not uniformly poor and focused on identify potential prognostic factors predicting survival in those patients who underwent ASCT after achieving a CR (65). Investigators stratified patients with *TP53-*mutated AML/MDS according to the hematopoietic cell transplantation specific comorbidity index (HCT-CI), performance status (PS), and disease status at the time of transplant and individualized a distinctive group of patients who showed significantly better outcomes after the procedure. Patients presenting more than one risk factor in the analysis showed inferior long-term survivals and should be enrolled in new trials including novel therapies before and/or after ASCT in order to reduce the potential risk of relapse and prolong survival after the procedure.

## Functional Classification of *TP53* Mutation in AML

It is not yet elucidated whether distinct types of *TP53* mutations (non-sense, missense, deletions, insertions, slice site mutations) produce a uniformly dismal outcome in AML. Several studies have demonstrated that there are no differences in terms of OS and EFS between AML patients with missense *TP53* mutations and those with truncating aberrations and that GOF activities elated for some missense *TP53* variants do not represent the crucial mechanism of treatment resistance (66–69). A statistically significant impact on survival was identified between non-disruptive and disruptive *TP53* mutations in a large study including patients with head and neck cancers (70). “Non-disruptive mutations” are identified as mutations arising inside the L2 or L3 binding domains that determine a substitution of an aminoacid from the same polarity/charge category, or mutations outside the L2 or L3 binding domain (except stop mutations). “Disruptive mutations” are categorized as DNA hot-spot aberrations that produce a STOP-sequence determining a desegregation of *TP53* protein codification or DNA sequence alterations that arise inside the L2 or L3 binding domains (codons 163–195 or 236–251) and cause the substitution of an aminoacid with another of a different polarity/charge category. Conversely to what has been shown for solid tumors, discrimination into disruptive and non-disruptive *TP53* mutations does not indicate a different outcome in patients with AML (69). The Evolutionary Trace (ET) method is an extensively standardized approach to assess key functional or structural residues in proteins (71). A scoring system (EAp53) was developed in solid tumors (72) to categorize missense mutations of *TP53* gene into high- and low-risk alterations. Consequently, the same evolutionary action score (EAp53) has been also applied in AML patients to predict which types of *TP53* mutations are more deleterious. This algorithm includes mutations scoring between 0 and 100 with WT-*TP53* sequences holding a score of 0. In a recent study including a large cohort of 1,537 patients (AMLSG cohort) with newly diagnosed AML who received intensive treatments (69), the EAp53 score was applied to identify missense mutations and distinguish between low-risk (<75 mutations) and high-risk (≥75 mutations) groups (49 and 35 patients in the high-risk and in the low-risk group, respectively). However, utilizing a multivariate Cox proportional analysis a significant difference on OS and/or EFS was not detected (69). Recently, the Israeli group created a synthetic *TP53* mutation dataset and calculated the functional impact of more than 10,000 DNA-binding domain (DBD) *TP53* alterations in human cells in culture and *in vivo* (73). They proposed an algorithm based on the average of the relative enhancement or depletion of a specific *TP53* mutation detected at three different time points called “relative fitness score (RFS).” A high RFS identifies advantageous expansion in culture showing higher fitness of the variant, while a low RFS indicates advantageous depletion. In the AMLSG cohort a statistically significant superior OS (median 12.9 *versus* 5.5 months, *p* = 0.017) and a trend in longer EFS (7.3 *vs* 5.2 months, *p* = 0.054) were found for patients with a low-risk RFS comparing with those with a high-risk RFS (69). Therefore, the AML-specific RFS algorithm seems to hold a prognostic significance in *TP53*-mutant AML and to be a functional source for treatment decision-making.

## Treatment Options Beyond Standard Chemotherapy

Patients with mutant *TP53* AML who are treated with standard anthracycline-based and ARA-C-based induction regimens show poor outcomes, with post-induction response rates to standard therapeutic approaches amounting to 20–30% (37, 61). Furthermore, median survival in this setting range between 4 and 6 months, with an estimated OS after ASCT of roughly 10 months (37, 61). These unsatisfactory results clearly indicate that the dismal prognosis in patients with an unfavorable-risk cytogenetic profile, the presence of *TP53* mutations, or both may be crucial for the therapeutic approaches and may be mitigated with other potentially effective treatment modalities.

### Decitabine

Decitabine (DAC) (5-aza-2′-deoxycytidine) is routinely employed as monotherapy for treating patients with MDS and older patients with AML (74). Several studies have sought to identify biomarkers such as mutations and DNA methylation alterations in *IDH1*, *IDH2*, *TET2*, and *DNMT3A* that might predict responses to DAC (75, 76). Interestingly, recent clinical trials have demonstrated that patients with AML harboring *TP53* mutations displayed favorable responses to treatment with DAC (77, 78), despite the molecular mechanisms associated with DAC responses being currently unclear. Moreover, clonal assessments of patients treated with DAC showed the prominent, but not durable, clearance of sub-clones with *TP53* mutations (79). In a study, including 116 patients (26 with MDS) treated with a 10-day regimen of DAC in monthly cycles, Welch et al. reported high rates of complete morphologic response among *TP53* mutant patients (77). They observed a higher response rate in AML patients presenting an unfavorable-risk cytogenetic score compared with those stratified as intermediate-risk or favorable-risk according to cytogenetic analysis (67 *vs.* 34%). A higher response rate was also seen comparing patients with *TP53* mutations and patients with WT-*TP53* (100 *vs.* 41%). However, this study does not contribute to elucidate the exact mechanisms causing the marked sensitivity of patients with *TP53*-mutant AML or MDS to DAC. Further studies including 5-day regimens of DAC in monthly cycles reported 62 and 66% response rates in *TP53-*mutant AML or MDS patients, respectively (17, 80) and cell-line analysis also suggested a specific sensitivity of *TP53-*altered cells to hypomethylating treatment (81). Later, Chang et al. demonstrated in univariate and multivariate analyses that *TP53* mutations were the unique molecular abnormalities, which predicted a CR achievement after DAC in patients with MDS (78). Using routine immunohistochemistry (IHC), Ruzinova et al. also found that expression levels of *TP53* is associated with either *TP53* gene mutation profile or clearance in patients treated with DAC (82). Therefore, the authors postulated that *TP53* IHC may be used as a valid surrogate for assessing *TP53* mutation profile in patients with AML and may be specifically functional in cases for whom molecular testing cannot be applicable. Four studies (83–86), although they primarily included patients with MDS (532 of 550 patients, 97%), showed that patients with *TP53* mutations treated with various regimens of hypomethylating agents were characterized by response rates that were similar to, but not higher than, the rates observed among patients with WT-*TP53* (**Table 2**). In a single-center, open-label, randomized phase II trial, Short et al. assessed the efficacy of DAC given in either 5-day or 10-day schedules. There was no difference in response rates and outcomes between the two DAC schedules overall or when patients were stratified by cytogenetic risk, *de novo versus* t- or s-AML, or *TP53* mutation status. Authors observed several preliminary findings in this study that should be further evaluated in larger cohorts. First, they did not observe a correlation between baseline *TP53* VAF and response. In addition, they observed that the median *TP53* VAF at the time of remission was 8.4%, with four patients with a VAF of ≥20% at the time of remission, suggesting that some of these *TP53* mutations may be present in pre-leukemic clones and not exclusively in myeloblasts. Finally, they reported that the two patients in whom a *TP53* mutation could no longer be detected in the remission bone marrow had the longest durations of remission (87). Therefore, despite the current fervor for DAC, DAC as monotherapy does not seem to produce deep and durable responses in a *TP53*-mutated setting, and additional consolidation therapies appear to be necessary.

**Table 2:** Outcomes of patients with AML and MDS treated with hypomethylating treatments as single agent according to *TP53* mutation status.

Study	No^1^ of pts^6^	Pts^6^ with mutated *TP53* (%)	Type of treatment	Overall Response		p-value	Overall Survival		p-value
				Mutated *TP53*	Wild-type *TP53*		Mutated *TP53*	Wild-type *TP53*	
				No.^1^ of patients (%)			Median time (months)		
Welch et al. (43)	116 (90 AML^2^; 26 MDS^3^)	21 (18)	DAC^4^ 20 mg/m^2^ for 10 consecutive days in monthly cycles	21 (100%)	32 (41%)	<0.001	12.7	15.4	0.79
Bejar et al. (47)	213 (213 MDS^3^)	39 (18)	42 (20%) pts^6^ received AZA^5^; 144 (68%) DAC^4^; 27 (13%) DAC^4^ plus another medication	20 (51)	80 (46)	NR^7^	Hazard ratio for death, 2.01 (95% CI 1.29–3.14)		0.002
Bally et al. (46)	62 (18 AML^2^; 44 MDS^3^)	23 (37)	AZA^5^ 75 mg/m^2^ for 7 consecutive days in monthly cycles	10 (43)	20 (51)	0.6	12.4	23.7	<0.001
Jung et al. (49)	107 (107 MDS^3^)	13 (12)	66 (62%) pts^6^ received AZA^5^; 41 (38%) DAC^4^	10 (77)	47 (50)	0.08	31% at 2 years	67% at 2 years	0.003
Chang et al. (45)	109 (109 MDS^3^)	15 (16)	DAC^4^ 20 mg/m^2^ for 5 consecutive days in monthly cycles	11 (73)	63 (67)	0.85	14	20	0.072
Takahashi et al. (48)	168 (168 MDS^3^)	38 (23)	38 (23%) pts^6^ received AZA^5^; 40 (24%) DAC^4^; 79 (47%) DAC^4^ or AZA^5^ plus another medication; 11 (7%) guadecitabine	15 (39)	41 (32)	0.13	9.4	20.7	<0.001
Kadia et al. (22)	293 (293 AML)	53 (18%)	24 (45%) pts^6^ received DAC^4^ orAZA^5^; 11 (21%) high-dose cytarabine-based; 18 (34%) low-intensity therapy other than hypomethylating agents	7 (29%) in DAC^4^/AZA^5^ group	26/76 (34%) in DAC^4^/AZA^5^ group	0.3	8.7 (in pts^1^ >60 years old)	NR^7^	

^1^No, number; ^2^AML, acute myeloid leukemia; ^3^MDS, myelodysplastic syndrome; ^4^DAC, decitabine; ^5^AZA, azacitidine; ^6^ pts, patients; ^7^NR, not reported.

### Venetoclax

Venetoclax (VEN), a bcl-2 inhibitor, has displayed promising efficacy in combination with hypomethylating agents (HMAs) either in *de novo* or in relapsed/refractory (R/R) AML (81, 88, 89). Apoptosis mediated by VEN appears to be *TP53*‐independent (90, 91), and VEN/HMAs combination activity was reported in several high-risk leukemia genetic groups (81, 88, 89). In the trial that led to VEN approval by the FDA, encouraging remission rates were reported in patients harboring *TP53* mutations; in this setting, CR + complete remission with incomplete blood count recovery (CRi) rates was 47%, median duration of CR + CRi and median OS was 5.6 and 7.2 months, respectively (88). In a *post hoc* exploratory analysis assessing the baseline prognostic factors, *TP53* status figured as a significantly positive predictor for CR + CRi using univariate logistic regression models (92). These data suggested improvement compared with historical results that documented CR rates as low as 28%, although duration of response was short, consistent with those reported elsewhere (93, 94). Also, Aldoss et al. have reported that responses to VEN/HMAs combination strategies were encouraging not only in *de novo* AML, but also in the group of patients with R/R *TP53* mutated AML (CR/CRi rate 38%), which included also patients relapsing after prior ASCT (95). Furthermore, in this cohort, response rates were similar comparing patients who received either a 5‐ or a 10‐day schedule of DAC plus VEN and the VAF of *TP53-*mutated cases was comparable in responders and non‐responders, conversely to an MDS trial in which *TP53* VAF affected outcomes of patients treated with HMAs (96). The MDACC group reviewed 69 patients with AML and *TP53* mutations who received VEN-based regimens between 2014 and 2018 (52% in frontline and 48% in the salvage setting). The overall response rate (ORR) was 47 and 24% in *de novo* and R/R patients with AML, respectively; all six patients with negative MRD obtained a complete cytogenetic response after receiving VEN and maintained a CR for a median of 3.4 months (97) (**Table 3**). Recently, DiNardo et al. analyzed 81 patients with AML treated with VEN-based combinations in order to identify molecular correlates of durable remission, response followed by relapse (adaptive resistance), or refractory disease (primary resistance) (92). High response rates and durable remissions were typically associated with *NPM1* or *IDH2* mutations, with prolonged molecular remissions prevalent for *NPM1* mutations. Primary and adaptive resistance to VEN-based combinations were mainly correlated with two major mechanisms of resistance: activated kinase signaling and bi-allelic *TP53* perturbation. Using CRISPR/Cas9 to disrupt *TP53* function in AML cell lines, authors demonstrated that *TP53* loss favored therapeutic resistance to VEN, HMAs, and ARA-C alone as well as in combination. Notably, these data are strongly sustained by further preclinical reports which, using a genome-wide CRISPR/Cas9 screen in leukemic cell lines, showed VEN resistance associated with defects in apoptotic pathway members (*TP53*, *BAX*, and *PMAIP1*) (98, 99). Therefore, these findings suggest the need of surveying genetic *TP53* integrity both at diagnosis and in remission in order to identify patients likely to develop resistance to VEN-based therapy. The combination of VEN/HMAs seems to produce better outcomes compared with the poor prognosis reported in *TP53-*mutated AML patients who received combination treatments based on conventional chemotherapy (93, 94), indicating that improving response rates and remission duration may represent a safe bridge to transplantation for more patients.

**Table 3:** Outcomes of patients with AML treated with venetoclax combination strategies according to *TP53* mutation status.

Study	No^1^ of pts^6^	Type of AML^2^	Pts^6^ with mutated *TP53* (%)	Type of treatment	Overall Response	Duration of response	Overall survival
					No of patients (%)	Median time (months/range)	Median time (months/range)
Di Nardo et al. (64)	145	145 (100%) *de novo* AML^2^	36 (25)	73 (51%) pts^6^ received VEN^3^ plus DAC^4^; 72 (49%) VEN plus AZA^5^	17 (47)	5.2 (1.2–9.4)	7.2 (3.7–NR^11^)
Bejar et al. (47)	31	15 (49%) *de novo* AML^2^; 16 (51%) R/R^8^ AML^2^	31(100)	28 (90%) pts^6^ received VEN^3^ plus DAC^4^; 3 (10%) VEN^3^ plus AZA^5^	16 (52) 67% in *de novo* AML^2^ *vs.* 38% in R/R^8^ AML^2^ (*p* = 0.01)	NR^7^	NR^7^
Shoulker et al. (70)	69	36 (52%) *de novo* AML^2^; 33 (48%) R/R^8^ AML^2^	69 (100)	60 (87%) pts^6^ received VEN^3^ plus DAC^4^ or AZA^5^; 2 (3%) VEN^3^ plus FLAG-ida^9^; 3 (4%) VEN^3^ plus low dose Ara-C^10^; 5 (6%) VEN^3^ plus CPX-351	25 (36) 47% in *de novo* AML^2^ *vs.* 24% in R/R^8^ AML^2^	6.4 months in *de novo* AML^2^ *vs.* 3.6 months in R/R^8^ AML^2^	3.6 months in *de novo* AML^2^ *vs.* 2.5 months in R/R^8^ AML^2^

^1^No, number; ^2^AML, acute myeloid leukemia; ^3^VEN, venetoclax; ^4^DAC, decitabine; ^5^AZA, azacitidine; ^6^pts, patients; ^7^NR, not reported; ^8^R/R, relapsed/refractory; ^9^FLAG-ida, fludarabine, cytarabine, G-CSF, idarubicin; ^10^ARA-C, cytarabine; ^11^NR, not reached.

## Other Therapeutic Perspectives

Alternative approaches are ongoing to treat the ultra-high-risk class of patients with *TP53*-mutated AML. To date, AML with *TP53* mutations lacks a clinically standardized therapeutic strategy aimed at targeting these alterations. The primary therapeutic end-point in this setting should be to restore normal *TP53* functions, reactivating intact *TP53* activities. However, multiple clinical trials are ongoing with the aim of selectively targeting mutant *TP53*. Recently, small molecules have been developed with the specific abilities of destabilizing individual *TP53* point mutants (100). These are exciting, targeted therapies although their effectiveness might be challenging to evaluate in AML clinical trials due to the small number of patients with target mutations (**Table 4**).

**Table 4:** Ongoing clinical trials targeting *TP53* mutant and wild-type AML.

Target	Clinical trial number	Phase	Trial status	Small molecule	Antineoplastic combination
**Mutant TP53**	NCT03072043	IB/II	Recruiting	APR-246	Azacitidine
**MDM2**	NCT01773408 NCT02098967 NCT02545283 NCT03671564 NCT02319369 NCT03634228 NCT03041688 NCT02143635	I/IB I III I I I/II IB I	Completed Completed Recruiting Recruiting Recruiting Recruiting Recruiting Recruiting	Idasanutlin/Cometinib Milademetan AMG-232 HDM201	Cytarabine Alone or with Azacitidine Cytarabine Decitibine
**MDM2 and BCL-2**	NCT02670044	IB/II	Recruiting	Dasanutlin/Venetoclax	
**MDM2 and MDMX**	NCT02909972	I	Recruiting	ALRN-6924	Alone or Azacitidine
**HMG-CoA reductase**	NCT03560882	I	Recruiting	Atorvastatin	
**Several targets**	NCT03381781	II	Recruiting	Arsenic Trioxide	Decitabine

### Potential Molecularly Targeted Therapies for p53-Mutant Cases

Several approaches, including inactivation of mutant p53, degradation of mutant p53, and restoration of the wild type function of p53, have been studied. Overall, two crucial approaches have been used to target mutant *TP53* in treatment of human cancers. The first approach includes small agents able to directly target mutant p53 by induction of its degradation or reactivation of its tumor-suppressive transcriptional activity (101). Among compounds that induce degradation of mutant p53 there are agents such as Hsp90 inhibitors [reverse the Hsp90’s function to inactivate MDM2 and Hsc70‐interacting protein (CHIP)] (102), statins (induce CHIP-dependent degradation of p53 with conformational changes) (103), or HDAC inhibitors (inhibit HDAC6 and disrupt the HDAC/Hsp90/mutant p53 complex) (104). Among compounds that restore wild-type function of p53 there are agents such as CP-31398 (stabilize the DNA-binding core domain and induce conformational changes) (105), PETIC (sensitize the p53 mutant to proteasome-mediated degradation and restore p53 WT conformation) (106), RITA (reactivate p53 in mutant p53 cancers by inhibiting the p53-HDM2 interaction) (107), and COTI-2 (restore WT p53 activity by targeting and binding to misfolded p53 mutant) (108). The alternative approach is to targeting pathways that are critical for the survival and growth of p53 mutant cancers. Specific molecular targets include G2/M regulators (CHK1/2, MK2, PLK2) (109–111) kinases (SGK2, MPS1) (112, 113) and growth pathways (p38, DAPK1) (114, 115). This latter approach is justified by the fact that, over the last years, several molecules was identified to be critical for survival or growth of cells with p53 mutations that can be targeted for the selective treatment of p53-mutant cancers (116, 117). Ongoing efforts continue to identify such critical mutant p53-specific survival and growth regulatory pathways. Therefore, novel drugs that target mutant p53 or the critical pathways activated by p53 mutation are highly promising for effective treatment of many cancers, including AML and probably these agents represent the future landscape in AML scenario.

### APR 246

Pharmacologic strategies able to stabilize both WT and mutant *TP53* promoting the reactivation of tumor-suppressor activities are under study in AML. The most encouraging is APR-246, a pro-drug converted to methylene quinuclidinone (MQ), which links through a covalent bond the mutant *TP53* core domain, promoting the increased regulation of transcriptional factors involving in apoptotic pathways (116, 117). Therefore, cells acquire functions promoting apoptotic mechanisms and cell cycle arrest pathways. The first human trial including the use of this agent showed a reduction of blast percentage from 46 to 26% in the bone marrow of the unique patient enrolled in the study with AML harboring a *TP53* core domain mutation (118). In a recent phase Ib/II trial, APR-246 was associated with 5-azacitidine (5-AZA) for the treatment of *TP53*-mutated MDS and AML patients. Interestingly in 11 evaluable patients, the combination of these agents provided an ORR of 100% and a CR rate of 82%. Moreover, the majority of remissions were characterized by deep molecular responses showing a median VAF of 0.3% in NGS-negative patients (119). The efficacy of APR-246 as maintenance therapy after ASCT in AML and MDS patients is currently being assessed in phase II trials. A phase I dose-finding and cohort expansion trial is also ongoing to assess the safety and preliminary efficacy of APR-246 combined with VEN and 5-AZA in patients with myeloid malignancies (NCT04214860).

### Immunotherapeutic Approaches

Checkpoint inhibitors function inhibit co-receptors on T-cells, such as PD-1 and CLTA-4, which play a role in immune evasion by cancer cells. Augmented expression of these receptors and their ligands have been detected on leukemic blasts, with further upregulation observed after treatment with HMAs (120). This suggests a possible resistance mechanism to HMA therapy and provides rationale for combination therapy. Notably, increased expression of PD-L1 has been identified in blasts and hematopoietic stem cells in *TP53* mutant patients in comparison to wild-type patients (121). A recent phase 2 study of nivolumab in combination with 5-AZA in R/R AML showed tolerable toxicity and an ORR of 33%, although higher response rates were observed in HMAs-naïve patients and those with increased bone marrow and peripheral blood T-cells by flow cytometry (122). *TP53* mutations were found in 23% of patients and mutation status did not appear predictive of response. Preliminary results from trials assessing checkpoint inhibitors in combination with HMAs, in combination with induction chemotherapy and as post-remission maintenance treatment in MDS and AML patients have recently been presented demonstrating tolerability and promising early efficacy data, with numerous trials ongoing (123–129). Macrophages represent a crucial cell type involving in the innate immune response with CD47 consisted in a dominant macrophage checkpoint. CD47 has a key role in signaling pathways and appears overexpressed in myeloid malignancies leading to tumor evasion of phagocytosis by macrophages. Inhibition of CD47 induces engulfment of leukemic cells and their potential therapeutic elimination (130). Pre-clinical data has showed encouraging anti-cancer activity in several hematologic diseases including AML and MDS. Magrolimab, a CD47 inhibitor, was assessed in a phase 1b study either as single agent in R/R AML/MDS or in association with 5-AZA in untreated AML patients non-eligible for intensive chemotherapy and untreated MDS patients who are stratified as intermediate, high, or very high risk according to the Revised International Prognostic Scoring System (IPSS-R) (131). Forty-six AML/MDS patients were efficacy evaluable. In AML patients, 14/22 (64%) showed an objective response with 55% achieving a CR/CRi. In MDS 22/24 (92%) patients responded with 50% achieving a CR. The MRD negativity was seen in 57 and 23% in AML and MDS responders, respectively. Magrolimab + 5-AZA efficacy was also investigated in AML patients with *TP53* mutations. The CR/CRi rate in 9 *TP53* mutant AML patients was 78% with 44% achieving CR and 33% achieving CRi. In addition, MRD negativity was observed in 57% of responders and median duration or median survival was not reached with a median follow-up of 6.9 months. *TP53* mutant VAF appeared substantially decreased or eliminated in all patients (131).

### *MDM2* Inhibitors

Recent results demonstrate that several AML subgroups can develop *TP53* dysfunction due to the occurrence of different events, such as *NPM1* and *FLT3* mutations, *MDM2/MDMX* upregulation, fusion proteins promoting chromosomal reciprocal translocations, and aberrant expression of specific miRNAs (132). *MDM2* inhibition is a promising target in the treatments of AML, serving as a negative regulator of *TP53*; the activation of *TP53* functions induced by any stimuli or DNA damages up-regulates transcription of *MDM2* mRNA and codification of protein that in turn links *TP53* and directly decreases or inhibits its activity through multiple mechanisms (132). The first *MDM2* inhibitor tested into human clinical trials was RG7112 (133). In AML scenario, RG7112 was assessed either as single agent (133) or combined with low-doses of ARA-C (134). Some patients even reached CR after having RG7112 and received transplant. RG7388 (idasanutlin) is a second-generation *MDM2* inhibitor that resulted more selective and potent, and hold a better pharmacodynamics/pharmacokinetic profile when it was compared with RG7112. Furthermore, this agent produced dose-dependent *TP53* stabilization, apoptosis, and cell cycle arrest (135). In a multicenter phase I/Ib trial, idasanutlin was assessed in AML patients either as single agent (5-day schedule of idasanutlin in monthly cycles) or in association with ARA-C (1 g/m^2^ IV for 5 days every 28 days) at escalation doses (136). Seventy-six patients were enrolled in the combination arm; specifically, 23 and 21 patients in the dose escalation and dose expansion cohorts, respectively, while 32 patients in a bridging cohort (137). The CR rate was 25% (n = 19); the composite CR rate [composite CR (cCR), CR + CR with incomplete platelet recovery (CRp) + Cri] was 29%. In order to define biomarkers able to predict response, MDM2 protein expression on peripheral blood leukemic blasts and HSPC was measured by flow cytometry. A grater *MDM2* expression both on leukemic blasts and on HSPC was strictly correlated with CR; *TP53* mutational status alone was not. These data highlight the significance of *MDM2* expression in leukemic blasts identifying it as a positive predictive factor for disease remission. Preclinical studies in *TP53* wild-type AML tumor models have also shown synergism between idasanutlin and bcl2-inhibitors, such as VEN and ABT-263 (navitoclax) (138, 139). The bcl-2 inhibitors may induce apoptosis in G1 compartments while idasanutlin may determine G1 arrest causing nuclear fragmentation in the G1 phase of the second cycle (140). A phase I/Ib study including >60 years old patients with R/R AML who are not eligible for intensive chemotherapy is now ongoing to evaluate the efficacy of idasanutlin and VEN combination (NCT02670044).

### Other Agents

Arsenic trioxide (ATO) in association with ATRA is the standard combination therapeutic approach for low and intermediate-risk acute promyelocytic leukemia (APL) patients (141). It has been reported that ATO is able to inactivate *TP53* functions *via* the 26S proteasome pathway (142). Furthermore, ATO amplifies WT-*TP5*3 functions and indices the upregulation of its target genes, thereby promoting apoptosis and inactivating the proliferation of neoplastic cells (143). In a recent *in vitro* study, leukemic blast cells deriving from patients with AML and APL were tested with graded doses of ascorbate (ASC), alone or in combination with standard concentration (1 μM) of ATO. The ASC/ATO combination was enabled to induce myeloid blasts degradation, including leukemic CD34^+^ cells, sparing CD34^+^ progenitors deriving from bone marrow and normal cord blood (144). The pro-apoptotic effect of ASC/ATO treatment seemed to be associated with augmented oxidative stress and reactive oxygen species (ROS) overexpression. Furthermore, recent studies identify a statin (atorvastatin), a cholesterol-lowering drug, as a degradation inducer for conformational or misfolded *TP53* mutants with minimal effects on WT *TP53* and DNA contact mutants (103).

## Conclusions

*TP53* mutations characterize a distinctive setting of AML associated with recurrent karyotypic aberrations, the lacking of recurrent single nucleotide mutations, dismal outcomes, and scarce responses to intensive therapeutic regimens. However, the role of *TP53* mutations in AML is enigmatic. Conversely to several other human cancers, a great majority of AML display no genomic *TP53* alterations. There is now growing appreciation of the fact that the unaltered *TP53* status of tumor cells can be exploited therapeutically. Therefore, the use of pharmacological activators of the *TP53* pathway may provide clinical benefit in AML. The importance of detecting *TP53* mutations in all AML age categories is justified by the fact that the presence of *TP53* mutations contribute to a very high risk of treatment failure with standard chemotherapy approaches and thus *TP53* status is an important consideration for designing novel therapeutic strategies for AML. In patients with *TP53* mutations, these alterations may lead to novel, selective vulnerabilities, creating opportunities for therapeutic targeting of *TP53* mutant AML. DAC has been reported as a potential efficient approach, being characterized by a *TP53*-independent mechanism of action. Some studies showed that the survival rate among AML patients with unfavorable-risk cytogenetic profiles, *TP53* mutations, or both and are treated with DAC is comparable to that of patients who have an intermediate-risk cytogenetic profile and receive DAC. However, further studies are required to verify whether these differences in survival are truly due to improved responses associated with DAC or to decreased survival rate of patients with unfavorable-risk cytogenetic profiles treated with conventional chemotherapy including anthracycline and ARA-C. The combination of VEN/HMAs seems also to give some benefit in this setting, improving response rates and remission duration and potentially representing a safe bridge to transplantation for selected more patients. Unconventional strategies and *TP53-*targeted therapeutics are now being tested as monotherapy or in combination with conventional drugs in order to further improve the response rate especially in R/R AML patients and to increase the number of patients potentially eligible for transplantation, which remains the only curative option for these patients. A wide armamentarium of small-molecule activators of the *TP53* pathway, and an increasing understanding of molecular pathways triggered by mutant *TP53* have accelerated efforts aimed at targeting *TP53* function in AML. In combination with standard AML chemotherapy or emerging targeted therapies, pharmacological targeting of the *TP53* pathway may provide therapeutic benefit in AML. Further therapeutic efforts and novel targeted therapeutic options will be necessary in order to overcome the unfavorable risk related to *TP53* mutations.

## Author Contributions

MM, CM, and PN wrote the manuscript. PF critically revised the paper and approved the final version. All authors contributed to the article and approved the submitted version.

## Conflict of Interest

The authors declare that the research was conducted in the absence of any commercial or financial relationships that could be construed as a potential conflict of interest.

## Abbreviations

AEL, acute erythroid leukemia; AML, acute myeloid leukemia; APL, acute promyelocytic leukemia; ASC, ascorbate; ASCT, allogenic stem cell transplantation; ATO, Arsenic trioxide; CLL, chronic lymphocytic leukemia; CMML, chronic myelomonocytic leukemia; CR, complete response; cCR, composite complete remission; Cri, complete responses with incomplete blood count recovery; CRp, complete remission with incomplete platelet recovery; DAC, decitabine; DBD, DNA-binding domain; DFS, disease free survival; DNR, daunorubicin; EBMT, European Society for Blood and Marrow Transplantation; EFS, event free survival; ELN, European Leukemia net; ET, evolutionary trace; GOF, gain of function; HCT-CI, cell transplantation specific comorbidity index; HMAs, hypomethylating agents; HSPC, hematopoietic stem/progenitor cells; IHC, immunohistochemistry; IWG-PM, International Working Group for Prognosis in MDS; LOH, loss of heterozygosity; MDACC, MD Anderson Cancer Center; MDS, myelodysplastic syndromes; MQ, methylene quinuclidinone; MRC-AML, myelodysplasia-related changes acute myeloid leukemia; MRD, minimal residual disease; NGS, next generation sequencing; NRM, non relapse mortality; OS, overall survival; PEL, pure erythroid leukemia; PS, performance status; R/R AML, relapsed/refractory acute myeloid leukemia; ROS, reactive oxygen species; t-AML, therapy related acute myeloid leukemia; TCGA, the Cancer Gene Atlas; t-MDS, therapy related myelodysplastic syndromes; VAF, variant allele frequency; VEN, venetoclax; WT-TP53, wild-type TP53; 5-Azacitidine, 5-AZA
